# Mesh penetrating the cecum and bladder following inguinal hernia surgery: a case report

**DOI:** 10.1186/s13256-017-1435-8

**Published:** 2017-09-14

**Authors:** Hiroshi Asano, Saori Yajima, Yoshie Hosoi, Makoto Takagi, Hiroyuki Fukano, Yasuhiro Ohara, Nozomi Shinozuka, Takaya Ichimura

**Affiliations:** 1 Department of General Surgery, Saitama Medical University, 38 Morohongou Moroyama Irumagun, Saitama, 350-0495 Japan; 2 Department of Pathology, Saitama Medical University, 38 Morohongou Moroyama Irumagun, Saitama, 350-0495 Japan

**Keywords:** Inguinal hernia, Kugel method, Mesh penetration, Cecum, Bladder

## Abstract

**Background:**

Tension-free repair using mesh is a common inguinal hernia surgical procedure. However, various complications such as mesh-related infection and recurrence may develop as a result. Moreover, although rare, there are also reports of intestinal obstruction caused by adhesion of the mesh to the intestinal wall and cases of mesh migration into various organs. Here, we report our experience with a patient in whom mesh extraction was performed due to migration of mesh into the intestinal tract following inguinal hernia surgery and formation of a fistula with the bladder.

**Case presentation:**

Our patient was a 63-year-old Japanese man who had a history of operative treatment for right inguinal hernia during early childhood. Because a relapse subsequently occurred, he was diagnosed as having recurrent right inguinal hernia at the age of 56 years for which operative treatment (the Kugel method) was performed. He presented to our hospital 6 years later with the chief complaint of lower abdominal pain. Computed tomography findings revealed a mass shadow in contact with his bladder and cecal walls, and enteric bacteria were detected in his urine. Furthermore, because lower gastrointestinal endoscopic findings confirmed mesh in the cecum, we performed operative treatment. The mesh had migrated into the cecum and a fistula with his bladder had formed. We removed the mesh through ileocecal resection and partial cystectomy.

**Conclusions:**

It appeared that a peritoneal defect occurred when the mesh was placed, allowing the mesh to migrate into our patient’s intestinal tract. Because contact between the mesh and the cecum resulted in inflammation, a fistula formed in his bladder. It is important to completely close the peritoneum when placing the mesh.

## Background

Tension-free repair using mesh has become a widely utilized technique in inguinal hernia surgery because it has a lower relapse rate and fewer postoperative complications than other procedures [1]. However, mesh-specific complications have also been reported, including mesh infection and migration [2]. When the mesh comes into contact with the organs of the digestive tract or elsewhere, rigid adhesions can occur, causing intestinal obstruction and migration of the mesh into the internal organs.

Chuback *et al*. [3] reported a case of small bowel obstruction caused by mesh migration into the abdominal cavity following curative hernia surgery with a mesh plug, and Murphy *et al*. reported a case of a sigmoid colonic fistula secondary to a mesh plug [4]. Also, there have been cases of mesh migration into organs other than those of the intestinal tract, such as the bladder [5].

Normally, the peritoneum is between the mesh and the abdominal cavity, so the mesh cannot come into direct contact with the intestines or other organs. However, mesh migration is believed to occur because of a defect in the peritoneum due to incomplete peritoneal repair [6] or because of peritoneal damage due to excess tension from the mesh. Various surgical techniques used to repair the inguinal hernia have been reported in association with mesh migration [7, 8], but there have been no previous reports of mesh migration following use of the Kugel method.

The present report describes a case of mesh migration into the intestinal tract complicated with bladder fistula that occurred 6 years after surgical repair using the Kugel method.

## Case presentation

Our patient was a 63-year-old Japanese man who had a history of operative treatment for right inguinal hernia during early childhood, although the type of procedure was unknown. Relapse subsequently occurred, and at the age of 56 years, he received a diagnosis of a recurrent right inguinal hernia, for which the Kugel method was performed. Six years later, he visited a local physician with the chief complaint of lower abdominal pain. Computed tomography (CT) showed thickening of his bladder and cecal wall; mesh infection was suspected and he was referred to our department.

**Fig. 1:**
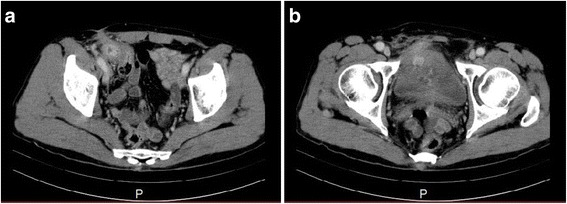
Abdominal computed tomography findings. **a** A poorly defined mass is observed to be in contact with the cecum. **b** The mass is also in contact with the bladder wall

**Fig. 2:**
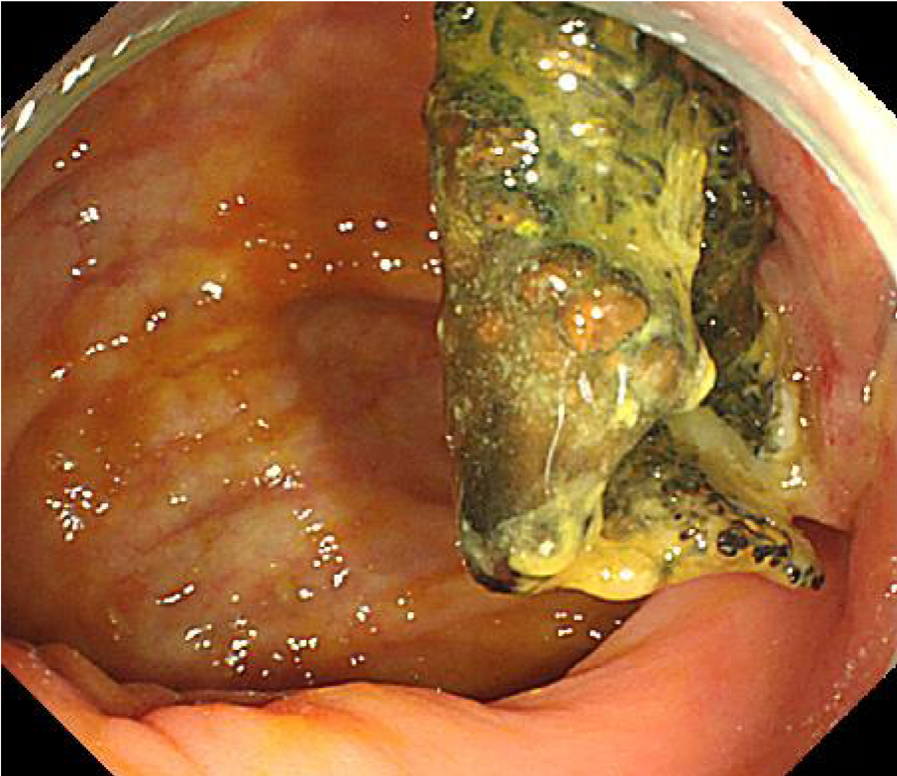
Lower gastrointestinal endoscopic findings. Mesh migrating through the cecal wall on the contralateral side of the Bauhin valve

Mild tenderness was apparent in his lower abdomen at presentation. Hematologic findings revealed that although his white blood cell count was normal, his C-reactive protein was slightly increased to 1.44 mg/dl. His urine analysis was positive for occult blood and bacterial contamination, and enteric bacteria were detected in urine cultures. An abdominal CT showed a poorly defined mass in contact with the cecal wall in the right inguinal region; the mass was also in contact with his bladder wall (Fig. 1). When lower gastrointestinal endoscopy was performed, a Kugel patch that had migrated into his intestinal tract was confirmed in the cecum wall on the side opposite to the Bauhin valve (Fig. 2).

Because we suspected that the fistula formation in his intestinal tract and bladder was due to the mesh, we scheduled a mesh removal procedure, but our patient felt left testicular pain 2 days before surgery, and an ultrasound examination revealed swelling of the left epididymis. Therefore, we diagnosed epididymitis. We thought that a mesh-related urinary tract infection was the cause of the epididymitis and performed surgical removal of the mesh.

**Fig. 3:**
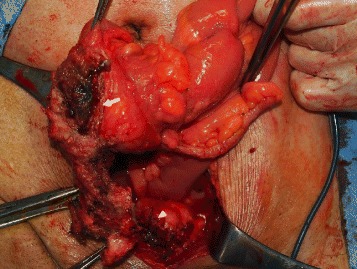
Intraoperative findings. The mesh is migrating into the cecum (*arrow*) and the bladder (*tip of arrow*)

Following laparotomy with a midline abdominal incision in which intraperitoneal observation was performed, the cecum was found to be firmly adhered near the right internal inguinal ring. There was no mesh exposure in his abdominal cavity, but when the adhesion was separated, we confirmed the presence of mesh migrating into his intestinal tract and piercing the cecal wall from the preperitoneal space. The inner side of the mesh was in contact with his bladder wall. We performed ileocecal resection to extract the mesh, and the mesh, including bladder wall, was finally excised by partial resection (Fig. 3). Partial cystectomy usually involves the insertion of a urethral catheter to reduce pressure; however, as the cause of epididymitis was chronic urinary infection, there were concerns that the insertion of a urethral catheter could prolong urethritis. Hence, a temporary cystostomy was established. In addition, 2 g/day cefmetazole sodium was administered to treat epididymitis from the day before until 1 week after surgery.

**Fig. 4:**
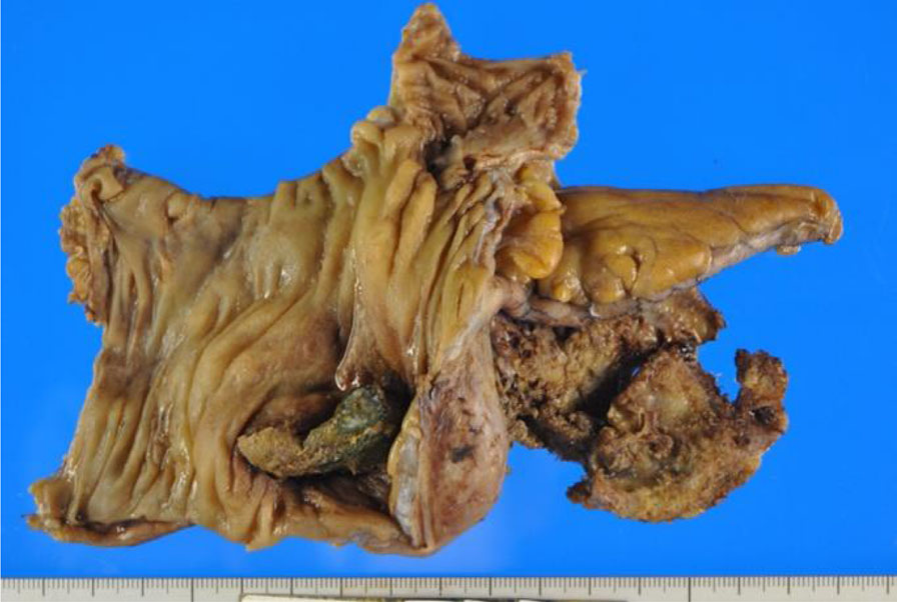
Excised specimen. The cecal wall has been penetrated and the mesh is exposed in the intestinal tract

**Fig. 5:**
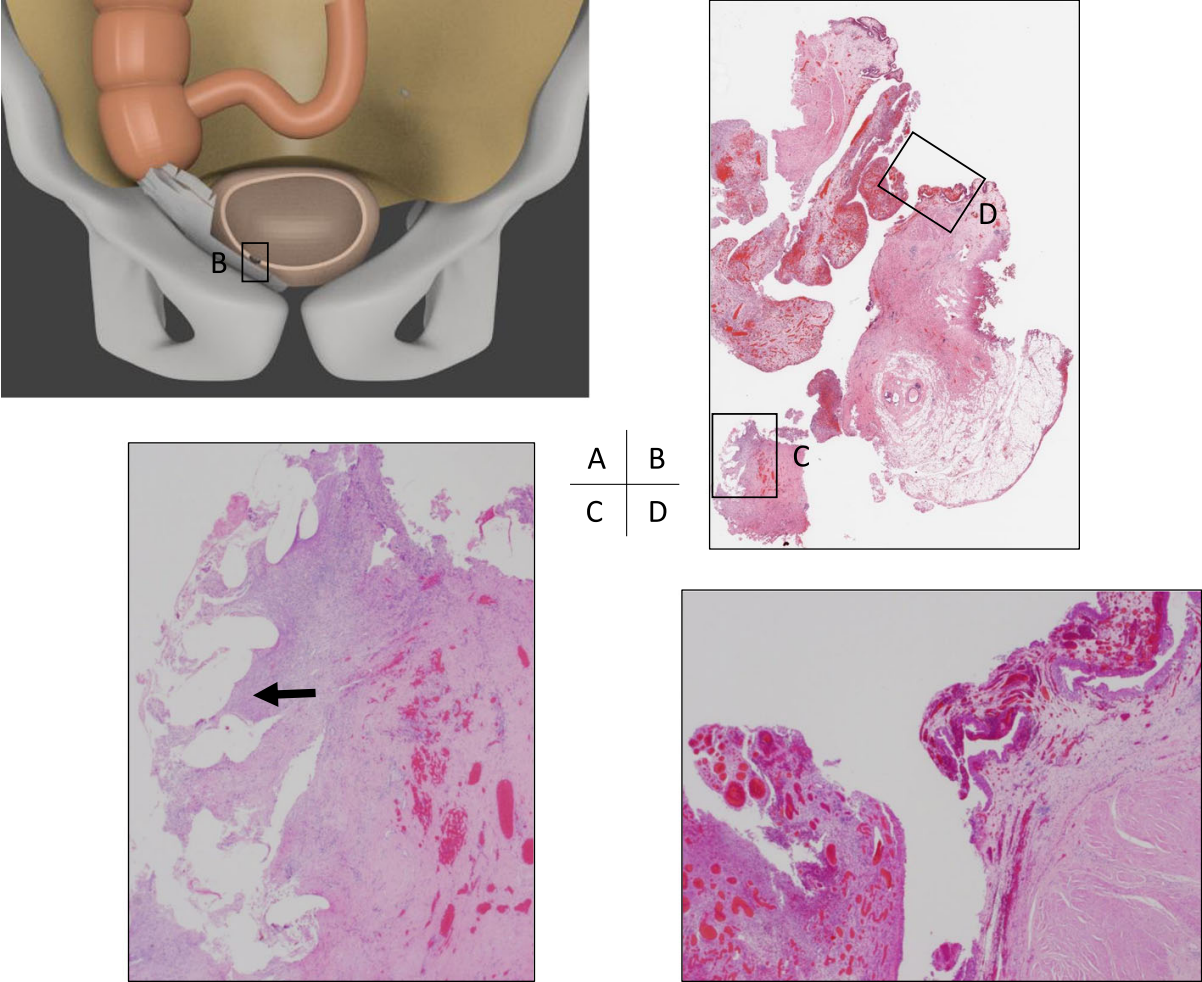
Histopathological findings. **a** The positional relationship between the mesh and the organs is shown. **b** Loupe image of the fistula. Mesh traces are observed in the fibrous portion of fat surrounding the bladder (*arrow* **c**). Fistula formation (**d**) is observed in the bladder lumen continuously from this site, but the mesh did not penetrate further than the muscle layer

In the excised specimen, the mesh had penetrated the cecal wall and was exposed in the intestinal tract (Fig. 4). On histological examination, a trace of mesh was found in the fibrotic area around the urinary bladder. The mesh did not penetrate into the muscularis propria; however, an inflammatory fistula was found between the trace of mesh and the lumen of the urinary bladder (Fig. 5). Postoperative wound infection occurred, but subsequently improved with drainage. The cystostomy was closed on postoperative day 11. After confirming the disappearance of the abscess cavity, our patient was discharged on postoperative day 38.

## Discussion

When inguinal hernia surgery is performed, the tension-free method is standard, but operative techniques such as the plug and Kugel method utilizing various meshes are also performed. However, as the use of mesh has become more common, mesh-specific complications such as intestinal obstruction and migration of the mesh into the bladder have been reported [9–11]. A search of PubMed for organ migration identified reports concerning the plug method, the Prolene Hernia System (PHS) method [7], and the Lichtenstein method [8], but we could not confirm any reported cases of migration following the Kugel method, which was utilized in the present case. Organ migration generally occurs to a single organ such as the bladder, cecum, or sigmoid colon, and there is only one previous reported case in which complications simultaneously occurred in two organs [12], as in the present patient.

Direct contact between the mesh and the organs [13] and strong tension on the organs [4] has been implicated as the mechanism of mesh migration into the internal organs or fistula formation following inguinal hernia surgery. Displacement due to insufficient fixation can also result in migration [9]. Because the mesh is conventionally placed in the preperitoneal space, direct contact between mesh and intestinal tract is impossible because of the presence of the peritoneum. However, if closure of the peritoneum at the high ligation is incomplete or if damage to the preperitoneal space is overlooked, it causes the mesh to come into contact with the intestinal tract. In addition, a plug with a characteristic shape featuring a convex tip, and the Kugel patch, which autonomously widens through a shape memory ring, may both cause strong tension on surrounding organs depending on the location of placement.

In the case of the present patient, there were adhesions around the hernia sac caused by inguinal hernia surgery during early childhood, and it is possible that peritoneal closure was incomplete. Moreover, because the mesh had migrated so as to protrude into the intestinal lumen, it is possible that the border of the mesh may have caused excessive strain on the intestinal wall. Thus, it can be assumed that the mesh and the cecum came into contact with each other, causing a foreign body reaction, and postoperatively, the mesh gradually migrated into his intestinal tract over time due to movement of the mesh and intestinal tract [14, 15].

As to the fistula formation with his bladder, there is a possibility that the mesh and his bladder wall came into contact, similar to what happened in the intestinal tract. However, the Kugel patch is normally placed so as to be in contact with the bladder wall, and the tissue intervening between them is the preperitoneal fat; the peritoneum is not present. Nevertheless, there are almost no reports in which the Kugel patch formed a fistula with the bladder, and it is unlikely that only contact between the mesh and the bladder caused the fistula in the present case. Furthermore, because in the present patient a trace of mesh was found in the fibrosis site around his bladder, and a fistula covered with inflammatory granulation tissue was present in the bladder lumen at this site, it can be postulated that the fistula was formed because of the infection caused by the mesh after it migrated.

In the present patient, approximately 6 years had passed since the mesh was placed, and it is unknown when the migration occurred. A review of the literature on mesh migration revealed that symptoms develop from 1 to 20 years after surgery [5, 15]. If the cause of migration is related to the surgical procedure, such as contact between the mesh and organs or excessive tension due to the mesh, postoperative symptoms may develop early, but in cases in which symptoms develop after several years have passed, it appears that not only changes from the surgical procedure but also changes in mesh shape and positional relationship with organs over time are significant factors.

## Conclusions

The frequency of migration of mesh to the internal organs and fistula formation is low, but it is difficult to treat when the condition occurs. Although there are various causes, in terms of the surgical technique, in order to prevent complications it is important to completely close the peritoneum when placing the mesh and sufficiently separate the preperitoneal space so as to not allow the mesh to place tension on the peritoneum and surrounding organs.

## Abbreviations

CT: Computed tomography
PHS: Prolene Hernia System
